# Phylogenomics and Plastome Evolution of Tropical Forest Grasses (*Leptaspis, Streptochaeta*: Poaceae)

**DOI:** 10.3389/fpls.2016.01993

**Published:** 2016-12-27

**Authors:** Sean V. Burke, Choun-Sea Lin, William P. Wysocki, Lynn G. Clark, Melvin R. Duvall

**Affiliations:** ^1^Department of Biological Sciences, Northern Illinois University, DeKalbIL, USA; ^2^Plant Tech Core Unit, Agricultural Biotechnology Research Center, Academia SinicaTaipei, Taiwan; ^3^Department of Ecology, Evolution, and Organismal Biology, Iowa State University, AmesIA, USA

**Keywords:** Anomochlooideae, Pharoideae, plastome phylogenomics, plastid genome, Poaceae

## Abstract

Studies of complete plastomes have proven informative for our understanding of the molecular evolution and phylogenomics of grasses. In this study, a plastome phylogenomic analysis sampled species from lineages of deeply diverging grasses including *Streptochaeta spicata* (Anomochlooideae), *Leptaspis banksii*, and *L*. *zeylanica* (both Pharoideae). Plastomes from next generation sequences for three species were assembled by *de novo* methods. The unambiguously aligned coding and non-coding sequences of the entire plastomes were aligned with those from 43 other grasses and the outgroup *Joinvillea ascendens*. Outgroup sampling of grasses has previously posed a challenge for plastome phylogenomic studies because of major rearrangements of the plastome. Here, over 81,000 bases of homologous sequence were aligned for phylogenomic and divergence estimation analyses. Rare genomic changes, including persistently long ψ*ycf1* and ψ*ycf2* loci, the loss of the *rpoC1* intron, and a 21 base tandem repeat insert in the coding sequence for *rps19* defined branch points in the grass phylogeny. Marked differences were seen in the topologies inferred from the complete plastome and two gene matrices, and mean maximum likelihood support values for the former were 10% higher. In the full plastome phylogenomic analyses, the two species of Anomochlooideae were monophyletic. *Leptaspis* and *Pharus* were found to be reciprocally monophyletic, with the estimated divergence of two *Leptaspis* species preceding those of *Pharus* by over 14 Ma, consistent with historical biogeography. Our estimates for deep divergences among grasses were older than previous such estimates, likely influenced by more complete taxonomic and molecular sampling and the use of recently available or previously unused fossil calibration points.

## Introduction

Grasses (Poaceae) dominate terrestrial ecosystems that account for approximately 40% of the land area of the earth (Gibson, 2009). The most conspicuous of these ecosystems are open habitats, such as pampas, prairies, steppes, veldts, and alpine grasslands. In spite of the dominance of grasses in contemporary open habitats, grass systematists demonstrated some time ago that the family originated as species adapted to the understories of tropical forests (Clark et al., 1995). In phylogenetic analyses, descendants of these deep grass lineages form a grade of three subfamilies: Anomochlooideae, Pharoideae, and Puelioideae (Grass Phylogeny Working Group [GPWG I], 2001; Grass Phylogeny Working Group [GPWG II], 2012; Soreng et al., 2015, and many other references). All other grass species, here referred to as the crown group clade (CGC), form a major radiation sister to Puelioideae. Of the 12 subfamilial lineages, the most deeply diverging are Anomochlooideae and Pharoideae.

Anomochlooideae comprise two genera of perennial lowland forest grasses both found in tropical America. Species in this group are the most morphologically divergent in the family. *Anomochloa marantoidea* Brongn., the only species in this genus, has a particularly distinctive reproductive morphology. Lodicules, typically found in grass florets are absent and stamens number four, rather than the more usual (1–) 3 (-6) found in other species. Bracts and secondary axes in the inflorescence are in atypical positions and no structures with clear homologies to glumes, lemmas, or paleas are present (Sajo et al., 2008, 2012). Because of their unique structure, inflorescence units in this species are interpreted as spikelet equivalents (Clark and Judziewicz, 1996; Sajo et al., 2008; Kellogg, 2015) rather than the true spikelets characteristic of other grasses. This distinction is the basis for the name “spikelet clade,” which refers to all other Poaceae (11 other subfamilies and 11,000 species) excluding Anomochlooideae (Grass Phylogeny Working Group [GPWG I], 2001). *Streptochaeta* Schrad. ex Nees, the other genus in the subfamily, has three species. The florets of species in this genus have a somewhat more typical number of six stamens. The inflorescence units are similarly interpreted as spikelet equivalents. Lodicules are homologous to tepals, but at least in one species of *Streptochaeta* there are alternative tepal homologs, which are three distalmost bracts in the spikelet equivalent (Preston et al., 2009). Molecular data suggested that the ancestor of *Anomochloa* and *Streptochaeta* diverged prior to the divergence of all other groups of grasses (Clark et al., 1995; Mathews et al., 2000; Grass Phylogeny Working Group [GPWG I], 2001; Givnish et al., 2010). However, the uncertain homologies of their unique floral organs with those of grasses led Morris and Duvall (2010) to suggest that the affinities of Anomochlooideae with Poaceae should be reevaluated.

Pharoideae are also perennial grasses of tropical and subtropical forests (Watson and Dallwitz, 1992 onward). The genera divide into two groups: (1) *Leptaspis* R. Br. and *Scrotochloa* Judz. are biogeographically and morphologically similar. Both are paleotropical, although the range of *Leptaspis* extends further west into Madagascar and Africa and east to New Caledonia^1^. Lodicules in the two genera are small or absent. Both have female spikelets with urceolate (urn-shaped) lemmas open only at an apical pore. These similarities have caused some authorities to synonymize *Scrotochloa* with *Leptaspis* (Clayton and Renvoize, 1986)^2^ although others recognize the two as separate genera (Watson and Dallwitz, 1992; Soreng et al., 2015; World Checklist of Selected Plant Families (WCSP), 2015) and still others find the affinities among the pharoid genera ambiguous (Kellogg, 2015). (2) The second group comprises the single genus, *Pharus* P. Browne, which is a biogeographically and morphologically distinct genus. *Pharus* is exclusively neotropical. Lodicules are restricted to staminate flowers, when present, and the lemmas of female spikelets are unusual in form: cylindrically shaped with the margins enrolled against the palea.

In previous studies, complete plastid genomes (plastomes) of Pharoideae and Anomochlooideae were characterized by surveying specific types of mutations that often marked single divergence points in phylogenies. These events, which are called rare genomic changes (RGC) in recent studies of grass plastomes (Jones et al., 2014; Duvall et al., 2016; Orton et al., 2016), are distinguished not only by relative infrequency, but can also be attributed to mutational processes that distinguish them from microstructural changes such as non-reciprocal site-specific recombinations (Graur and Li, 2000). Morris and Duvall (2010) presented a detailed comparison of plastome structure in *Anomochloa marantoidea* to those of other grasses. Several features relating to two pseudogenes and an intron were atypical for the plastomes of other grasses, which may also be present in *Streptochaeta*, but this has not been confirmed with published sequence data. Complete characterization of these features in the plastomes of both genera in the subfamily requires full plastome data for *Streptochaeta*. Jones et al. (2014) used plastome phylogenomics to confirm the topology of deeply diverging Poaceae. They were also able to systematically document RGCs that marked specific branch points in the phylogeny. Finally, they estimated divergence times among these deep lineages finding that the lower ends of these ranges extended to considerably older dates than previously estimated.

In this paper, we extend the studies of Morris and Duvall (2010) and Jones et al. (2014), adding complete plastomes from three species of the most deeply diverging subfamilies of grasses. To date, no phylogenomic analyses of complete plastomes have been published that include representatives of both paleotropical and neotropical Pharoideae and both genera of Anomochlooideae. *Pharus* and *Leptaspis* have previously been included together in few phylogenetic studies: one morphological analysis by Kellogg and Campbell (1987) and two multi-gene analyses of three or four plastid loci (Christin et al., 2013; Jones et al., 2014).

Exploring the significance of these early diverging lineages in Poaceae is facilitated when the sampling can be balanced with other ingroup taxa and non-grass outgroups for comparison. At this writing there are considerably more complete plastomes from ingroup Poaceae than from any other angiosperm family^3^. However, outgroup sampling poses a challenge for plastome phylogenomics of grasses. Multiple overlapping inversion events, some of which are 10’s of kilobases in size, have variously rearranged the large single copy (LSC) regions in grasses and other Poales compared to those of all other angiosperms (Doyle et al., 1992; Katayama and Ogihara, 1996; Michelangeli et al., 2003; Wysocki et al., 2016). The difference in plastome synteny means that the use of non-grass outgroups is problematic. For example, part of the LSC region that is approximately 30,000 base pairs (bp) in size no longer aligns unambiguously between grasses and their outgroups. This study will employ methods to use an outgroup plastome and preserve as much homologous sequence as possible, without falsely representing the evolutionary history. For the outgroup, the recently published *Joinvillea ascendens* plastome (Wysocki et al., 2016) was chosen due to its close relationship to Poaceae (Prasad et al., 2005; Marchant and Briggs, 2007; Vicentini et al., 2008; Jones et al., 2014; Soreng et al., 2015).

Extensive plastome phylogenomic analyses of CGC grasses are reported in published studies (Wu et al., 2009; Wu and Ge, 2012; Burke et al., 2014; Cotton et al., 2015; Saarela et al., 2015; Duvall et al., 2016). These show that the approach offers good resolution with generally robust support values and allows for more confident estimates of divergence dates. Previous studies of complete grass plastomes have investigated partitioning the plastome in different ways (e.g., major plastome subregions or coding and non-coding sequences) to remove signal that conflicts with the dominant phylogenetic signal. Partitioning studies failed to show clear advantages when restricting the plastome data beyond the removal of ambiguously aligned regions (Zhang et al., 2011; Burke et al., 2012; Ma et al., 2014; Cotton et al., 2015; Saarela et al., 2015). The use of all unambiguously aligned coding and non-coding sequences from the entire plastome in these studies substantially increased phylogenetic information and raised support values to their maximum levels at most nodes. In this study, we apply a similar approach, with a focus on Anomochlooideae and Pharoideae.

Here we investigated the complete plastomes of two pharoid and one anomochlooid species, which were newly sequenced, and analyzed them with 43 other grass plastomes and one outgroup to address three objectives. (1) Given some of the unresolved questions of synonymy and intergeneric relationships in Pharoideae, we assessed whether *Leptaspis* and *Pharus* were reciprocally monophyletic. (2) We identified molecular evolutionary events, particularly those that could be considered RGC, in a plastome phylogenomic context. In particular, we determined whether specific RGC such as pseudogenizations and alterations in the length of protein coding sequences (CDS) previously observed in *Anomochloa* and *Pharus* were conserved across their respective subfamilies. (3) We extended the time-tree analysis of Jones et al. (2014). Here we added complete plastomes from over 40 species to our analysis and used more fossil calibration points, including a recently published root node fossil (Poinar et al., 2015). Divergence dates were estimated for the same species in parallel from a two gene matrix and the complete plastome matrix to contrast previous analytical approaches with the full plastome phylogenomic approach employed here. With these improvements we explored the divergence of neotropical from paleotropical Pharoideae and explored the time of origin of the divergence of the two genera of Anomochlooideae.

## Materials and Methods

### Sampling

Sampling from the three deeply diverging lineages of grasses included two species of Anomochlooideae, four species of Pharoideae, and one species of Puelioideae (four of these were previously sequenced and published). Three complete plastomes, followed by their voucher, collection location, and subfamily, were newly sequenced for this study including *Streptochaeta spicata* Schrad. Ex Nees [L. Clark and D. Lewis, 1642 (ISC); Brazil; Anomochlooideae], *Leptaspis banksii* R.Br. [S.-H. Wu, 139796 (HAST); Southern Taiwan; Pharoideae], and *Leptaspis zeylanica* Nees ex Steud. [L. Attigala, 156 (ISC); Sri Lanka; Pharoideae].

### Next Generation Sequencing

Plastome sequences were obtained using two different next generation sequencing (NGS) methods to accommodate different starting quantities of DNA and available resources. (1) Genomic DNA was extracted from leaf tissue of *L*. *banksii* with the urea extraction buffer system following Sheu et al. (1996). A paired-end DNA library was prepared using the TruSeq sample preparation protocol following the manufacturer’s instructions (Illumina, San Diego, CA, USA). Sequencing was performed on a MiSeq instrument (Illumina, San Diego, CA, USA) at the NGS Core facility at Academia Sinica, Taipei, Taiwan. This method produced 300 bp fragments with an average insert size of 590 bp. (2) DNA was extracted from silica-dried leaf samples of *S*. *spicata* and *L*. *zeylanica* with the DNeasy Plant Mini Kit (Siege, Valencia, CA, USA) after homogenization of the tissues in liquid nitrogen. DNA extracts were diluted to a final concentration of 2.5 ng/μl in sterile water. Single-read DNA libraries were prepared with the Nextera protocol (Illumina, San Diego, CA, USA). The DNA Clean and Concentrator system was used for library purification (Zymo Research, Irvine, CA, USA). Sequencing was performed on the HiSeq2000 platform at the Iowa State University DNA Sequencing Facility, Ames, IA, USA. This method produced 100 bp reads. Sequencing techniques for each species are summarized (Supplementary Table 1).

### NGS Plastome Assembly and Verification

Illumina sequence reads from both the MiSeq and HiSeq instruments were assembled into complete plastid genomes with similar *de novo* methods. These methods are fully described in Wysocki et al. (2014). Briefly, assembly made use of the Velvet v. 1.2.08 software package (Zerbino and Birney, 2008). This process was iterative so that contigs from the previous Velvet assembly were reloaded for three additional assemblies. With each iteration, k-mer lengths were increased in steps of six from 19 to 85 bp. A second *de novo* assembly method was implemented for *L. banksii* in which SPAdes v. 3.5.0 (Nurk et al., 2013) was used to assemble reads specifying the same k-mer sizes. Contigs were scaffolded with the anchored conserved region extension method, which queries contig sets for sequences 20 or more bases in length that were found to be invariant across 75 Poaceae species. Any gaps remaining between contigs in the scaffolds were resolved with contigs or reads by locating perfectly overlapping regions of at least 20 bp on the end of an incomplete assembly. A final verification step was performed by mapping the quality-trimmed read pool against the *de novo* assembly using Geneious Pro v8.0.2 (Biomatters Ltd., Auckland, New Zealand). On inspection, any clear instances of erroneous assembly identifiable by low read depths or misaligned reads were manually repaired.

Assembly of the full circular plastome could not be completed in only one instance. This occurred in the *psbZ* – *psbM* intergenic spacer (IGS) of the *Streptochaeta spicata* plastome where there was no overlap of NGS reads. A reference alignment of the otherwise completely assembled plastome of *S. spicata* with that of *A. marantoidea* indicated a missing region estimated to be less than 1,200 bases in length. Two primers were designed from the sequence flanking this region: Strepto114F – CCACTAAACTATACCCGCCACATC and Strepto115R – CATAATCTCCAGCCCGTGAACTTAG. This region was PCR amplified and Sanger-sequenced following the methods of Dhingra and Folta (2005) as modified and described in Burke et al. (2014). The contig assembled from two Sanger-sequenced reads (one in each direction) substantially overlapped each other, had a minimum overlap of 450 bases with the flanking sequence, and completely spanned the missing region. The base composition of this region suggests a possible reason for the complete lack of read coverage. The region had an AT composition of over 88%, much higher than the overall plastome value of 62–64%, suggesting a procedural bias in the library preparation or sequencing techniques that relate to base composition.

### Plastome Annotation and Analysis

Fully assembled plastomes were annotated by alignment to a previously published and annotated plastome in Geneious Pro. For *S. spicata* the annotation reference was the complete plastome of *A. marantoidea* (NC_014062). For the two species of *Leptaspis*, the annotation reference was from *Pharus latifolius* (NC_021372). Reference annotations were transferred to the new plastome when there was a minimum shared similarity of 70%. CDS for each species were examined, and adjustments were made to correctly position locus boundaries, check for homologous pseudogenizations, preserve reading frames, and in the case of tRNAs and rRNAs to preserve full lengths. The endpoints of the large inverted repeat (IR) were located using the methods of Burke et al. (2012). BLASTn searches (Altschul et al., 1997) were conducted on selected sequence regions to compare identities with banked sequences.

### Phylogenomic Analyses

In this study we assembled a matrix from the three new species plus 43 other grass species and one outgroup species for phylogenomic analyses, taking advantage of the large number of complete plastomes available in Poaceae. This provided sampling from all subfamilies of grasses and ensured representation among the taxa associated with fossil calibration points for the divergence estimation analysis (see below). The 43 previously published species of Poaceae were: *Achnatherum hymenoides* (GenBank accession number: NC027464), *Ampelodesmos mauritanicus* (NC027466), *Anomochloa marantoidea* (NC014062), *Aristida purpurea* (NC025228), *Arundinaria gigantea* (NC020341), *Axonopus fissifolius* (NC030501), *Brachyelytrum aristosum* (NC027470), *Chionochloa macra* (NC025230), *Chloris barbata* (NC029893), *Danthonia californica* (NC025232), *Danthoniopsis dinteri* (NC030502), *Dichanthelium acuminatum* (NC030623), *Digitaria exilis* (NC024176), *Distichlis bajaensis* (NC029894), *D. spicata* (NC029895), *Elytrophorus spicatus* (NC025233), *Eriachne stipacea* (NC025234), *Greslania* sp. (KJ870993), *Hakonechloa macra* (NC025235), *Isachne distichophylla* (NC025236), *Leersia tisserantii* (NC016677), *Melica subulata* (NC027478), *Micraira spiciforma* (KJ920234), *Neyraudia reynaudiana* (NC024262), *Olmeca reflex* (KJ870997), *Olyra latifolia* (KF515509), *Oryza rufipogon* (NC022668), *Oryzopsis asperifolia* (NC027479), *Otachyrium versicolor* (NC030492), *Paspalidium geminatum* (NC030494), *Phaenosperma globosum* (NC027480), *Pharus lappulaceus* (NC023245), *Pharus latifolius* (NC021372), *Piptochaetium avenaceum* (NC027483), *Puelia olyriformis* (NC023449), *Rhynchoryza subulata* (NC016718), *Rottboellia cochinchinensis* (NC030615), *Sartidia dewinteri* (NC027147), *Setaria italica* (KJ001642), *Tenaxia guillarmodae* (NC029897), *Thyridolepis xerophila* (NC030616), *Thysanolaena latifolia* (KJ920236), and *Zea mays* (NC001666). The outgroup plastome was from *Joinvillea ascendens* (KX035098).

The matrix of complete plastomes was aligned in Geneious Pro with the MAFFT v6.814b plug-in (Katoh et al., 2005) using the auto function and other default settings. The inverted repeat region “A” (IRa) was excluded from the data matrix to avoid double representation of this large repeat. Because of the major rearrangements in the plastomes of Poales, over 30,000 bp of total plastome sequence would be lost in a direct whole plastome alignment of Poaceae and Joinvilleaceae. To preserve as much sequence as possible, 15 protein CDS in the gene-rich region of the LSC from *atpA* to *psbK* in both Poaceae and *Joinvillea* were extracted. Note that almost all of this region is lost in a standard alignment due to inversions. These CDS were rearranged into the same order, unambiguously aligned and concatenated onto the end of the sequence matrix. Sites for which gaps were introduced in at least one sequence by the alignments were excluded from the data matrix prior to phylogenetic analyses to remove ambiguously aligned regions.

A second matrix containing the same taxa was created with only the CDS from *ndhF* and *rbcL*. This was used to compare our results against a subset of older studies (Vicentini et al., 2008; Bouchenak-Khelladi et al., 2010, 2014), which analyzed more taxa, but with less molecular data. The two concatenated genes were aligned in Geneious Pro with the MAFFT v6.814b plug-in. Due to the limited amount of data, the gaps introduced by the alignment of the second matrix were not removed.

Maximum likelihood (ML) analyses (Felsenstein, 1981) were conducted with the GTR+G+I substitution model selected by jModelTest v2.1.3 (Guindon and Gascuel, 2003; Darriba et al., 2012) under the Akaike information criterion (Akaike, 1974) for both data matrices. The ML analyses were implemented in RAxML-HPC2 on XSEDE v8.2.8 (Stamatakis et al., 2008) at the CIPRES Science Gateway (Miller et al., 2010) to determine the ML tree and bootstrap values (ML BV). The substitution model was set to GTRGAMMA + I, bootstrapping was halted automatically under the autoMRE function, and all other parameters were default. Bootstrap consensus trees were generated using the Consense function of the Phylip software package v. 3.66 (Felsenstein, 2005).

Bayesian inference (BI) analyses were performed using MrBayes on XSEDE v3.1.2 (Ronquist and Huelsenbeck, 2003) at the CIPRES Science Gateway using the same substitution model as in RAxML (invgamma; nst = 6). Two independent MrBayes analyses were performed with four chains and two million generations each at which point the average standard deviation of split frequencies was <0.0075. Other parameters were set at defaults including the burn-in level of 25%. Finally SH tests were used to test each data matrix and the resulting best ML topology against that of the other data matrix.

### Estimation of Divergence Times

Divergence dates were estimated on the complete matrix of aligned plastomes and the two gene matrix. Evolutionary rate heterogeneity in grass phylogenetics is well documented (Christin et al., 2014), guiding our choice of the uncorrelated relaxed clock model implemented in BEAST v2.1.2 (Bouckaert et al., 2014). Parameters included the GTR substitution model with a gamma category count of six, an estimated shape parameter with an initial value of 0.91 for the complete matrix and 0.87 for the two gene matrix, and an estimated proportion of invariant sites with an initial value of 0.51 for both the complete and two gene matrix. Initial values were obtained from the ML analyses.

Relatively few of the described grass fossils are useful as calibration points either because of uncertainties in the age or the specific taxonomic identity of the fossil. The sampling here was designed to allow the use of eight fossil calibration points, six of which were reliably dated and diagnosed with extant homologous species (Vicentini et al., 2008; Iles et al., 2015). Two other fossil calibrations were also included. Jacobs and Kabuye (1987) described an African macrofossil identified as *L. zeylanica* based on the diagnostic characteristic of secondary veins that diverge from the midvein at an acute angle (Watson and Dallwitz, 1992), and excavated from the modern extant range. The 12.2 Ma age of the *Leptaspis* fossil was obtained from K/Ar dating. Secondly, a recently described macrofossil in Myanmar amber is that of a grass floret. The determination of this identification was based on grass spikelet reproductive morphology and grass-host specificity of ergot fungi. This fossil was stratigraphically dated to 97–110 Ma (Poinar et al., 2015).

Specific information regarding the eight fossils is given (**Table 1**). Priors were set to constrain relationships around the calibrated nodes in the topology generated by the ML plastome analysis. The calibration points were implemented as uniform distributions between the minimum age of the node, as constrained by the associated fossil, and the maximum age of the oldest known fossil for the family (110 Ma; Poinar et al., 2015), following the general methods of Christin et al. (2014).

**Table 1:** Information for calibration points used for the divergence estimation analysis.

Figure letter	Fossil	Fossil type/Evidence	Age/Lower Bound (Ma)	Assigned node^a^	Citations
A	Spikelet clade, unspecified	Spikelet in amber	97	Spikelet clade	Poinar et al., 2015
B	Oryzeae	Phytoliths	66	Oryzoideae	Prasad et al., 2011
C	*Stipa florissanti*	Fruits	34	Stem node Stipeae/Amplodesmeae	MacGinitie, 1953
D	*Leersia seifhennersdorfensis*	Inflorescence	30	(*Leersia, Oryza*)	Walther, 1974
E	*Distichlis* sp.	Leaf fragments	14	(*Distichlis spicata, D. bajaensis*)	Dugas and Retallack, 1993
F	*Leptaspis* cf. *zeylanica*	Leaf fragments	12	(*Leptaspis banksii, L*. *zeylanica*)	Jacobs and Kabuye, 1987
G	*Dichanthelium*	Fertile lemmas and paleas	8	(*Dichanthelium, Thyridolepis*)	Thomasson, 1978
H	*Setaria*	“Seeds”	7	(*Setaria, Paspalidium*)	Elias, 1942

*^a^All assigned nodes are crown nodes.*

Each BEAST analysis was conducted on the CIPRES Science Gateway for 40 million generations, logging at every 10,000 trees. Convergence was assessed with Tracer v1.6 (Rambaut et al., 2014). Trees were summarized with TreeAnnotator (Bouckaert et al., 2014) using a burn-in value of 25%. The complete plastome tree file was then imported into R (R Core Team, 2015) using the read.beast function in PHYLOCH (Heibl, 2008) to import the BEAST tree, and then visualized using the geoscalePhylo function in Strap (Bell Mark and Lloyd, 2014).

## Results

### Plastome Features

GenBank accession numbers for complete plastome sequences of *Streptochaeta spicata, Leptaspis banksii*, and *L. zeylanica* are KU666544 – KU666546, respectively. The two different methods of library preparation produced sequence files ranging from 7.1 to 12.3 million reads per species. *De novo* assemblies produced a mean of 2–14 contigs per plastome with minimum mean coverage of 44.7. Numbers of reads, assembled contigs, and mean coverage values for each plastome are reported (Supplementary Table 1). These are among the longest grass plastomes sequenced to date ranging from 141,811 to 148,609 bp in length (Supplementary Table 1). The overall structure in the newly sequenced plastomes was highly conserved and largely reflected the gene content, intron-exon structure, and gene order of the plastomes of other grasses. Within this conserved framework, RGC previously reported in the plastomes of *Anomochloa marantoidea* by Morris and Duvall (2010) and *Pharus* spp. by Jones et al. (2014) could now be reinvestigated to determine their degree of conservation. To this end, four specific regions were examined in the plastome of *Streptochaeta spicata*. (1) In the *trnN*(GUU) – *rps15* IGS of *S. spicata* a sequence of approximately 840 bases with a minimum nucleotide identity of 70% to *ycf1* of non-grass plant species was observed. An approximately 300 bp portion of this region was 96% identical to the ψ*ycf1* locus of *A. marantoidea.* (2) In the *rpl23* – *trnL*(CAA) IGS there is a region of 800 bases with a 97% nucleotide identity to the ψ*ycf2* locus of *A. marantoidea*. (3) The *rbcL* – *psaI* IGS of *S. spicata* is 1,967 bases in length and contains a full length ψ*rpl23* sequence of 108 bases, which is 96% identical to that of *Puelia olyriformis*. (4) The plastid *rpoC1* locus in *S. spicata* has an intron of 753 bases, which is 89% identical to that found in the plastome of *A. marantoidea*.

Five additional regions were investigated in the plastomes obtained from the two species of *Leptaspis*: (1) The *rpl33* locus in both species begins with a typical start codon, which is in frame with a downstream TAG stop codon and is a full length coding sequence of 201 bases. (2) Similarly, the *rps18* locus in both species begins with a typical start codon, which is in frame with a TGA stop codon and is 528 bases in length. So the *rpl33* and *rps18* loci exhibited characteristics of fully functional genes in these species. (3) The *rpoC1* loci of both species were co-linear with homologous loci in all grasses sequenced to date excluding species of Anomochlooideae. In other words, there was no evidence of the intron in these loci. (4) A 21 base insertion was found in the *rps19* loci of both species, which precedes the last two codons in the sequence, in the same position as in the two species of *Pharus*. The insertion differed by two substitutions between the two species of *Leptaspis* (ACGACGAGATTTMGTATCSTT). (5) The *atpB-rbcL* IGS of both species contained an inversion of 73 bases, which had 92.3% identity with the same inverted region in the two species of *Pharus*, and was five bases shorter. The length difference was due to a tandem repeat indel of TTCTA. This inverted region had 89.6% identity with the corresponding sequence region of *Puelia olyriformis*, which was not inverted.

### Phylogenomic Analyses

The plastome alignment length for the 47 species included 81,039 nucleotide sites. This was after removal of the IRa, the addition of all coding sequences from *atpA* to *psbK*, and then removing all sites with at least one gap introduced by the alignment. The exclusion of gapped sites removed inversions, other microstructural changes, and ambiguously aligned portions of the plastomes. The aligned data matrix and associated tree file is available at the TreeBase repository^4^.

For the plastome ML analysis, a tree was produced with a -lnL = 386488.14 (Supplementary Image 1). ML BV were 100% for all but two nodes, both of which were in the CGC: (1) An internal node in the PACMAD grasses uniting the group that was sister to the two species of Aristidoideae (ML BV = 52%). (2) A node uniting three species of Pooideae: *Ampelodesmos mauritanicus, Oryzopsis asperifolia*, and *Piptochaetium avenaceum* (ML BV = 92%). The BI analysis produced a tree (Supplementary Image 2) that was topologically identical to the ML tree. In the BI analysis, all posterior probability (PP) values were at maximum (PP = 1.0) except for the first node specified above for the ML analysis, which had an associated PP = 0.92.

The two gene alignment had a length of 3,753 bp and included the same 47 species from the full plastome alignment. The resulting ML analysis produced a tree with a -lnL = -21575.34 (Supplementary Image 3). The BI analysis produced a tree (Supplementary Image 4) with a similar topology. The one difference between the two trees was the location of *Digitaria exilis*, in Panicoideae (Supplementary Image 3). In both analyses, the PACMAD clade was resolved with maximum support as monophyletic but the BOP was not. The BOP clade was polyphyletic with Bambusoideae diverging first followed by Oryzoideae and finally Pooideae. In general, support values for the nodes of the two gene trees were lower than those of the respective complete plastome analysis. The mean BV for the complete plastome ML tree was 98.76% and that for the two gene ML tree was only 88.56%.

**FIGURE 1:**
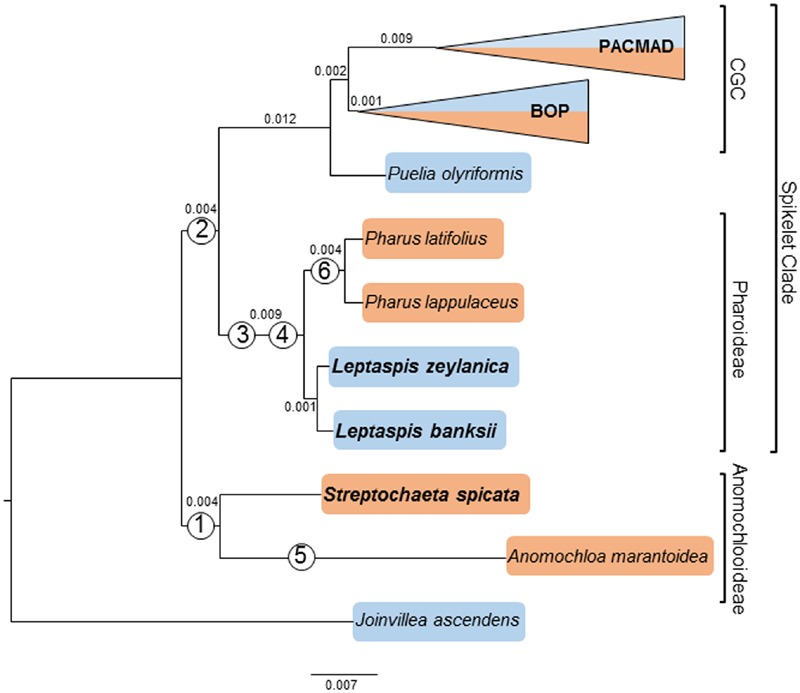
**Maximum likelihood phylogram inferred from 47 complete plastomes.** Branch lengths are proportional to the number of substitutions per site along the branch and are indicated on the branches. Newly sequenced species of Anomochlooideae and Pharoideae are emphasized with a larger font. The crown grass clade (CGC) and spikelet clades are indicated. Note that the Bayesian inference (BI) analysis produced a topology that was identical to the maximum likelihood (ML) topology. All depicted nodes are supported with 100% ML bootstrap values and PP = 1.0. The orange highlight indicates a New World modern distribution, while the blue indicates an Old World modern distribution. Numbers in circles identify six rare genomic changes (RGCs) denoted as: 1, large ψ*ycf1* and ψ*ycf2*; 2, loss of *rpoC1* intron; 3, *rps19* insertion; 4, *atpB-rbcL* intergenic spacer (IGS) 75 base inversion; 5, loss of ψ*rpl23*; and 6, ψ*rpl33* + ψ*rps18* dual pseudogenization.

Relationships within and between subfamilies of the CGC have been described in other plastome phylogenomic studies (Wu and Ge, 2012; Cotton et al., 2015; Saarela et al., 2015; Wysocki et al., 2015; Duvall et al., 2016). The phylogenomic results reported here will emphasize relationships among the two deeply diverging subfamilies, Anomochlooideae, and Pharoideae. The two species in Anomochlooideae were recovered as monophyletic, with maximum values in complete plastome analyses (ML BV = 100; PP = 1.0), and with high support in the two gene analysis (ML BV = 96; PP = 1.0). The four species of Pharoideae as a group were monophyletic with maximum support values (ML BV = 100; PP = 1.0) and *Pharus* and *Leptaspis*, with two species each, were also monophyletic with high support values (complete plastome: ML BV = 100; PP = 1.0; two gene: ML BV = 99; PP = 1.0). The six species in these two subfamilies were separated from the remaining grasses (*Puelia olyriformis* + CGC) by an internal branch in the ML tree with a length = 0.012 for the complete plastome and length = 0.021 for the two gene analysis. The branch subtending the four species of Pharoideae was of length = 0.009 for plastome matrix and a length = 0.017 for two gene matrix (**Figure 1**).

Finally, the SH test for the plastome matrix returned a significant result (*p* < 0.001) for the difference in the negative log likelihood for the two gene tree. The SH test for the two gene matrix did not return a significant result (*p* = 0.191) for the difference in the negative log likelihood compared to the complete plastome tree.

### Divergence Time Estimation

Two sets of somewhat different divergence estimations were obtained depending on the data matrix used. In the following, estimated times will be rounded to the nearest whole numbers, and the estimate obtained with the full data matrix will be listed first. For the 95% HPD intervals, the reader is referred to **Table 2**. The divergence times estimated for the deep branching events in Poaceae are of particular interest here. The estimated divergence of the spikelet clade from Anomochlooideae was 99 (101) Ma. Within Pharoideae, the two genera diverged from each other at estimated times of 40 (38) Ma. The two species of *Leptaspis* had an estimated divergence of 27 (17) Ma while those of the two species of *Pharus* were more recent at 12 (16) Ma. Within Anomochlooideae, *Anomochloa* was estimated to have diverged from *Streptochaeta* at 69 (70) Ma.

**Table 2:** Estimated divergence times for crown nodes together with the 95% highest posterior density (HPD) interval limits.

		BiStigmatic clade	Spikelet clade	Pharoideae	*Leptaspis*	*Pharus*	Anomochlooideae
With full plastome data set	Mean	90.87	98.90	40.43	26.93	12.31	68.67
	95% HPD upper	103.56	107.47	83.99	62.05	32.26	101.51
	95% HPD lower	81.70	88.01	15.83	12.10	1.04	28.20
With two gene data set	Mean	90.26	101.08	37.59	17.04	16.3	69.71
	95% HPD upper	97.45	106.66	61.76	26.35	31.12	100.37
	95% HPD lower	82.62	94.17	19.45	12.1	4.92	36.82

## Discussion

Plastome phylogenomic studies of the deeply diverging lineages of grasses require a suitable outgroup so that the deep topology can be determined and divergence times estimated. To date, there has been only one plastome-scale study of grasses and their outgroups, which was restricted to the analysis of CDS (Givnish et al., 2010). While interesting, this study was limited in term of total phylogenetic information and potentially affected by artifacts due to selection.

The history of multiple inversions in the plastome has caused major rearrangements across the Poales. Further complicating this history have been reinversions of partially overlapping segments, which restore the original order of some sets of loci, but leave other sets inverted so that the plastome becomes a complex and fragmented molecular mosaic. These rearrangements have hampered attempts to sequence complete plastomes of non-grass Poales. So until very recently there were no complete plastome sequences from families that are among the sister lineages to Poaceae to serve as an outgroup. The sequencing of a plastome from a species of Joinvilleaceae by Wysocki et al. (2016) presents an opportunity to further explore the divergence of deeply diverging lineages of grasses. Our approach here allows the use of complete IR and SSC region sequences and the portions of the LSC region that remain unaltered. Gaps that occurred due to the alignment were removed, and 15 ungapped coding sequences that were found in inverted regions were concatenated onto the remaining plastome data and realigned unambiguously preserving as much phylogenomic information as possible.

### Plastome Features

Determining the taxonomic extent of RGCs is valuable for the verification of branchpoints in phylogenies (**Figure 1**). Four previously described RGCs (Morris and Duvall, 2010; Jones et al., 2014) were found to be shared with other deeply diverging taxa. (1) The unusually large ψ*ycf1* and ψ*ycf2* loci previously found in *A. marantoidea* were found to be largely shared with similar loci in *Streptochaeta spicata*. Some interspecific differences between these loci were suggestive of degradation of the two pseudogenes along independent evolutionary paths after the two genera diverged, which we estimate at 69 (70) Ma. (2) The *rpoC1* intron, which is found in Joinvilleaceae and other non-grasses, is missing from almost all Poaceae. The *rpoC1* intron was not found in the two species of *Leptaspis* analyzed here, but was present in *S. spicata* as suggested by a previous, unconfirmed observation (Morris and Duvall, 2010). This means that both genera of Anomochlooideae have a species with this atypical characteristic that has been found in no other Poaceae to date and suggests that loss of the intron is a synapomorphy for the spikelet clade, but not the entire grass family. (3) A 21 base tandem repeat insertion in the coding sequence of *rps19*, which was originally described in two species of *Pharus*, is also found in two species of *Leptaspis*, suggesting that this repeat arose in a common ancestor. If *Scrotochloa*, the only other genus in Pharoideae, is sister to *Leptaspis*, as suggested by biogeography and reproductive morphology, then the *rps19* insertion is likely common across the subfamily. However, if *Scrotochloa* is sister to the remaining pharoid species, then this is not necessarily the case. At this writing there are no banked sequences from *Scrotochloa*. Further exploration of *rps19* loci in *Scrotochloa* and other species of *Leptaspis* and *Pharus* would determine the full extent of this *rps19* mutation. (4) An approximately 75 base inversion in the *atpB*-*rbcL* IGS was shared by the four species of Pharoideae. A two base IR sequence flanked the inversion, suggesting that the inversion forms the loop of a stem-loop structure.

Two RGCs were found to be restricted in taxonomic distribution to their original observed sampling. (1) In most Poaceae, the *rbcL* – *psaI* IGS is considered to be a hotspot for mutation. This IGS contains ψ*rpl23*, which is the hypothetical descendant of a non-reciprocal translocation of the functional *rpl23* locus from one of the IR regions (Katayama and Ogihara, 1996). The unusually short *rbcL* – *psaI* IGS of *A. marantoidea* was not shared with *S. spicata* or other species. Moreover, the latter species had a normal length ψ*rpl23* sequence. These observations suggest that this RGC is due to one or more autapomorphic deletions in *A. marantoidea*. (2) Jones et al. (2014) discovered simultaneous pseudogenizations in two loci, *rpl33* and *rps18*, which adjoin the same IGS and are found in two species of *Pharus*. The coincidental nature of the event that affected two adjacent loci suggested a common underlying cause, in this case probably one or more deletions. These observations in *Pharus* are unlikely to be due to sequencing method artifacts as the *P. latifolius* plastome was Sanger-sequenced while that of *P. lappulaceus* was sequenced with NGS methods. The *rpl33* and *rps18* loci in the two species of *Leptaspis* had typical start and stop signals in typical length reading frames. The apparently simultaneous pseudogenization of these two loci in the two species of *Pharus* is not a RGC that characterizes the entire Pharoideae and may actually be restricted to some subset of congeneric species in *Pharus*.

### Phylogenomics and Divergence Estimation

**FIGURE 2:**
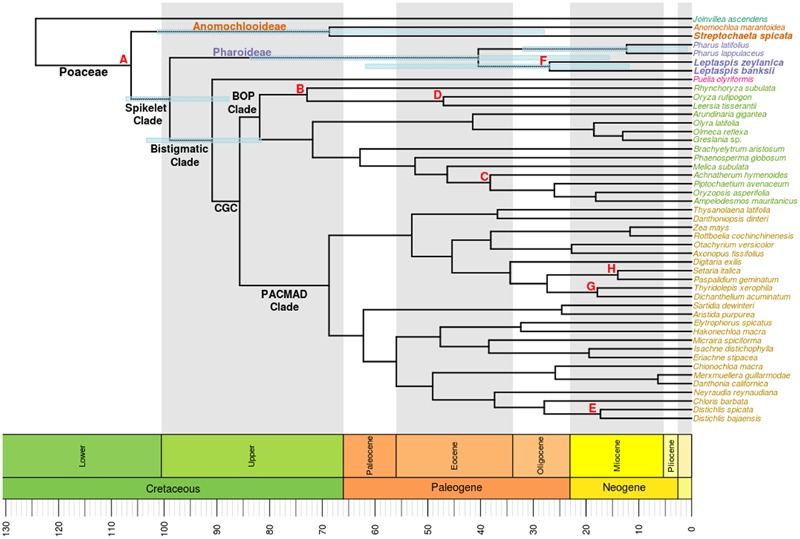
**Chronogram determined from divergence date estimation analysis using the complete plastome and the same species included in the tree of **Figure 1**.** Branch lengths are proportional to the amount of time since divergence, as indicated on the scale at the bottom of the figure. Fossil calibration nodes are represented with letters (**Table 1**). Bars illustrate the 95% highest posterior density (HPD) interval for clades of interest. Newly sequenced taxa are in bold.

Our plastome phylogenomic analyses with moderate taxon sampling typically show a high ratio of informative characters per node in phylogenetic trees and high bootstrap support values in grass phylogenies. The relatively long internal branches obtained in the deep phylogeny are suggestive of: (1) elevated substitution rates (Christin et al., 2014), (2) persistence through a long period, or (3) undocumented extinctions in the early history of the family. These are not mutually exclusive events and all may have contributed to the distribution of branch lengths in the deep topology of Poaceae. Our divergence estimation analyses incorporate three new aspects. (1) Our use of complete coding and non-coding plastome sequences more fully represents the historical signal in the plastid chromosome. Lower support values were observed in the two gene analysis compared to the whole plastome analysis, especially for more recent lineages in the CGC. The topological details of CGC lineages is beyond the scope of this study, but there was congruency and uniformly strong support observed for the deep branches in analyses of both matrices (**Figure 1**). The spikelet clade (Pharoideae + *Puelia* + CGC) was resolved here as monophyletic as in previous studies (e.g., Grass Phylogeny Working Group [GPWG I], 2001). Our estimated date of divergence of 99 (101) Ma for the spikelet clade predates other such estimates: ∼93 Ma (Bouchenak-Khelladi et al., 2010), ∼82 Ma (Prasad et al., 2005), 76 Ma (Jones et al., 2014), and 66 Ma (Vicentini et al., 2008). Similarly, our estimate for the divergence of the bistigmatic clade (*Puelia* + CGC) of 91 (90) Ma is older than other estimates: ∼80 Ma (Prasad et al., 2005), 67 Ma (Jones et al., 2014), and 56 Ma (Vicentini et al., 2008). While the divergence dates estimated from both the two gene and whole plastome data are similar, we suggest that some estimates are an artifact of using the limited subset of protein CDS. For example in the two gene analysis the divergence of Pharoideae and the species within *Leptaspis* and *Pharus* are estimated at 37, 17, and 16 Ma, respectively, while the complete plastome analysis recovered dates of 40, 26, and 12 Ma (**Figure 2**; **Table 2**). While the two estimated dates for Pharoideae were similar, the divergence of the species within each genus differed. This is most likely due to the lack of phylogenetic information of these two CDS (pairwise similarity in Pharoideae: 98.5%, *Leptaspis*: 99.4%, *Pharus*: 98.9%) when compared to the more variable and informative complete plastome sequences (Pharoideae: 95.2%, *Leptaspis*: 97.1%, *Pharus*: 98.7%). (2) Our analysis has a somewhat more complete representation of species among the deeply diverging grasses and at the same time includes an outgroup *J. ascendens*. (3) We also chose new fossil calibration points. The recently described grass macrofossil (Poinar et al., 2015) predates other fossils attributed to grasses yet is consistent in age with the diversification of grasses suggested by Indian phytolith fossils (Prasad et al., 2005, 2011). We also use the 12 Ma African macrofossil identified as *L. zeylanica* (Jacobs and Kabuye, 1987) as a lower bound for the divergence of the two species of *Leptaspis.* These fossils better calibrate an analysis of early diverging species.

The Anomochlooideae was recovered as monophyletic, with similar divergence dates from both analyses: 69 (70) Ma. The previously estimated dates of divergence for Anomochlooideae have been wide-ranging [∼76 Ma (Bouchenak-Khelladi et al., 2010), <10 Ma (Prasad et al., 2005), 65–104 Ma (Jones et al., 2014), and ∼57 Ma (Vicentini et al., 2008)]. Our estimate is comparable to that of Bouchenak-Khelladi et al. (2010). While their study did not analyze complete plastomes, it did include an older calibration, 90 Ma, for the minimum age of the crown group grass. This value was derived from other molecular estimates. Now there is fossil evidence that corroborates a similar age for the divergence of the oldest lineages of Poaceae.

The four species representing the pharoid subfamily formed a monophyletic group. *Pharus* and *Leptaspis* were reciprocally monophyletic, which is consistent with their allopatric distributions. The difference in age of divergences of the species of *Pharus* and *Leptaspis* suggests a scenario of historical biogeography. Poaceae likely originated in the Old World, as indicated by the location of the oldest grass macrofossil in Myanmar (Poinar et al., 2015) and the oldest grass phytoliths in India (Prasad et al., 2005). The deep divergence of Pharoideae in the grass phylogeny and the modern distributions of *Leptaspis* and *Scrotochloa* in the paleotropics suggests an Old World origin for the subfamily. All modern species of *Pharus* are neotropical, suggesting long-distance dispersal of the ancestor of this genus prior to its diversification. More recent divergences of the species of *Pharus* after dispersal to the New World is consistent with this scenario.

Our age estimate for Pharoideae suggests an origin at 40 (38) Ma, consistent with its long-term persistence as an independent lineage and/or the extinction of species on the branch leading to the Pharoideae. Other age estimates for this subfamily range from 44 to 71 Ma (Bremer, 2002; Jones et al., 2014). However, our age estimates for Pharoideae are the first, to our knowledge, to include molecular data from species of *Leptaspis*.

## Conclusion

The addition of new plastome sequences from two species of *Leptaspis* and one from *Streptochaeta* and the use of a recently sequenced outgroup plastome from *J. ascendens* allowed us to determine a more complete picture of the molecular evolution and ages of the deeply diverging subfamily lineages of Poaceae. During this early evolutionary period, RGCs accumulated in the plastomes so that they distinctly mark the divergences of Pharoideae and Anomochlooideae. Improved knowledge of the paleontological history of Poaceae and further molecular studies that incorporate nuclear phylogenomic data will continue to refine our knowledge of the early evolutionary events in this family.

## Author Contributions

MD and C-SL conceived the project design. C-SL, LC, and MD sequenced the specimens. SB and WW were involved with bioinformatic analyses. SB and MD conducted molecular evolutionary analyses and writing the manuscript. SB, C-SL, WW, LC, and MD were all involved with editing and finalizing the manuscript.

## Conflict of Interest Statement

The authors declare that the research was conducted in the absence of any commercial or financial relationships that could be construed as a potential conflict of interest.

The reviewer YS and handling Editor declared their shared affiliation, and the handling Editor states that the process nevertheless met the standards of a fair and objective review.

## Footnotes

1 http://apps.kew.org/wcsp/

2 www.theplantlist.org/tpl1.1/search?q=Scrotochloa

3 www.ncbi.nlm.nih.gov/genome/browse/

4 http://purl.org/phylo/treebase/phylows/study/TB2:S18781
