# Supplementary material for: Phylogenomics and Plastome Evolution of Tropical Forest Grasses (Leptaspis, Streptochaeta: Poaceae)
Source: Front Plant Sci. 2016 Dec 27;7:1993. doi: 10.3389/fpls.2016.01993 (PMC5186769; doi:10.3389/fpls.2016.01993)
Supplement: Supplementary file 1 [file Table_1.DOCX]

Supplementary Material

**Phylogenomics and plastome evolution of tropical forest grasses (*Leptaspis*, *Streptochaeta*: Poaceae)**

**Sean V. Burke^*^, Choun-Sea Lin, William P. Wysocki, Lynn G. Clark and Melvin R. Duvall**

**^*^Correspondence:** Sean V. Burke: [sburke5@niu.edu](mailto:mel-duvall@niu.edu)

**1 Supplemental 1**

Supplemental 1. Sequencing and assembly methods details, total and subregion lengths (nucleotides), and base composition for plastomes newly sequenced in this study.

| Species | Library  Preparation Method | Sequencing Method | Number of reads | Scaffolded contigs | Mean coverage | Total Length | LSC^b^ | SSC^b^ | IR^b^ | % AT |
| --- | --- | --- | --- | --- | --- | --- | --- | --- | --- | --- |
| *Streptochaeta spicata* | Nextera | Single end | 12,458,233 | 13 | 104.0 | 148,609 | 85,943 | 12,602 | 25,032 | 62.7 |
| *Leptaspis banksii* | TruSeq | Paired end | 8,854,085^a^ | 2 | 755.4 | 141,946 | 83,598 | 12,758 | 22,795 | 63.5 |
| *L. zeylanica* | Nextera | Single end | 7,121,151 | 14 | 44.7 | 141,810 | 83,188 | 12,770 | 22,926 | 63.4 |

^a^ Number of paired reads.

^b^ LSC = large single copy; SSC = small single copy; IR = inverted repeat.
