# Supplementary figures and images for: Phylogenomics and Plastome Evolution of Tropical Forest Grasses (Leptaspis, Streptochaeta: Poaceae)
Source: Front Plant Sci. 2016 Dec 27;7:1993. doi: 10.3389/fpls.2016.01993 (PMC5186769; doi:10.3389/fpls.2016.01993)

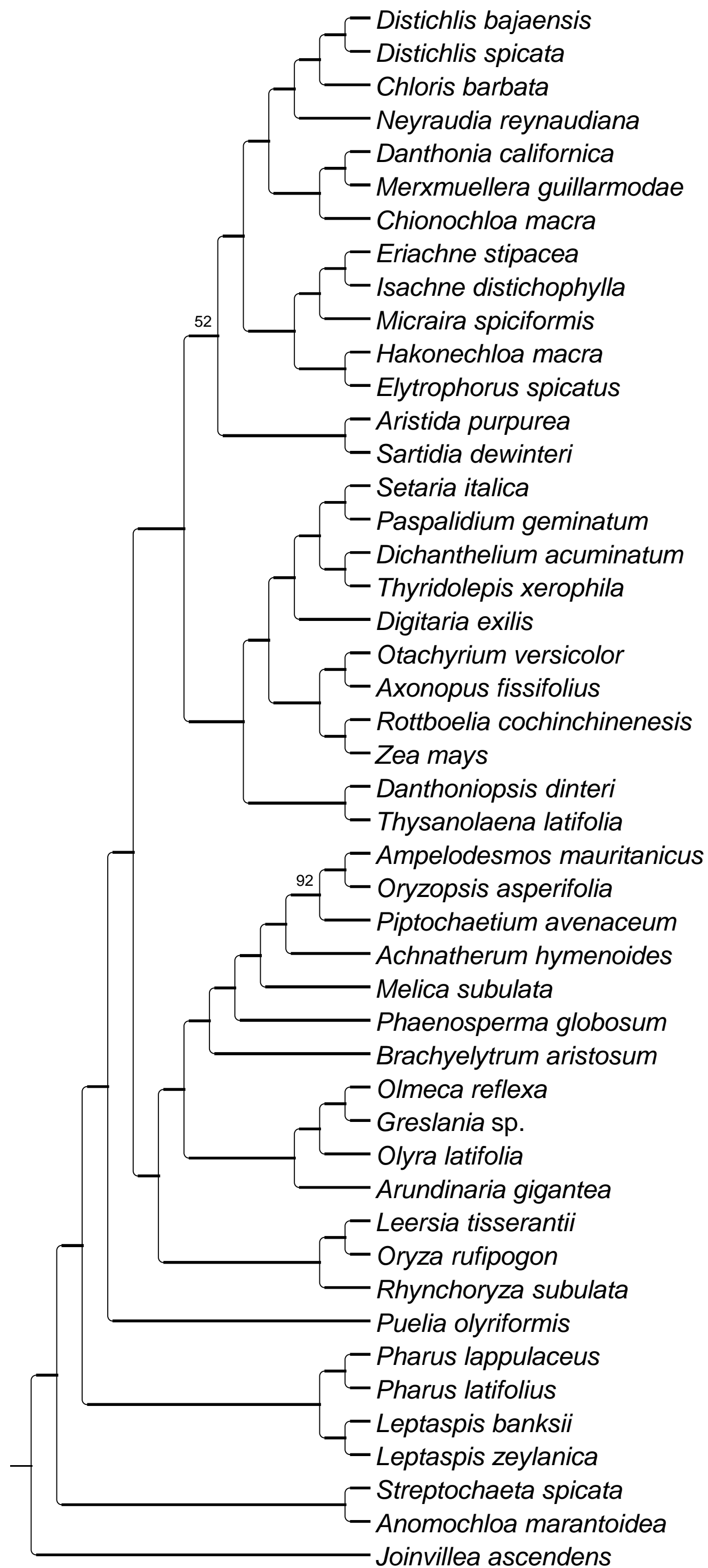

Supplemental 2: The ML tree from the complete plastome data set. All ML BV = 100% unless indicated.

Supplement: Supplementary file 2 [file Image_1.PDF]

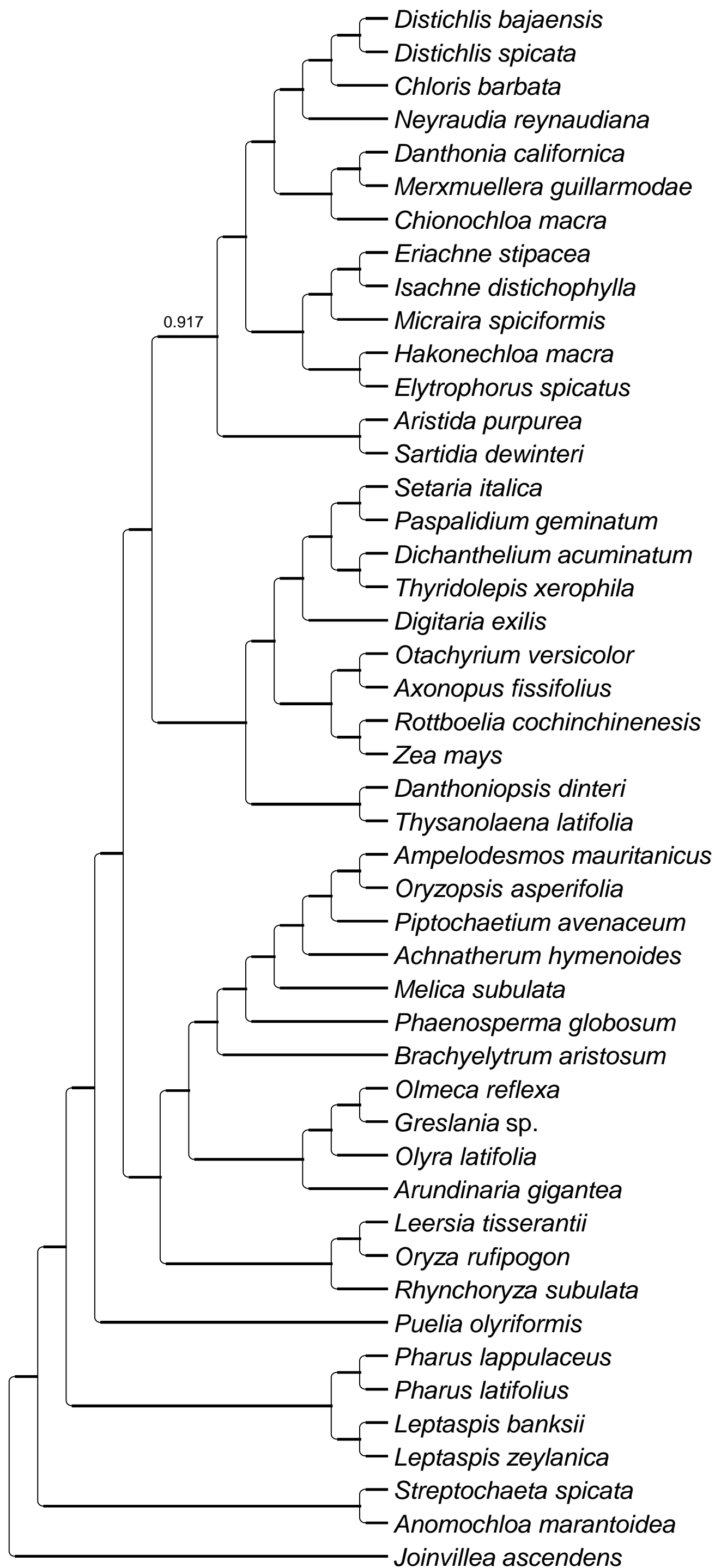

Supplemental 3: The BI tree from the complete plastome data set. All PP = 1.0 unless indicated.

Supplement: Supplementary file 3 [file Image_2.PDF]

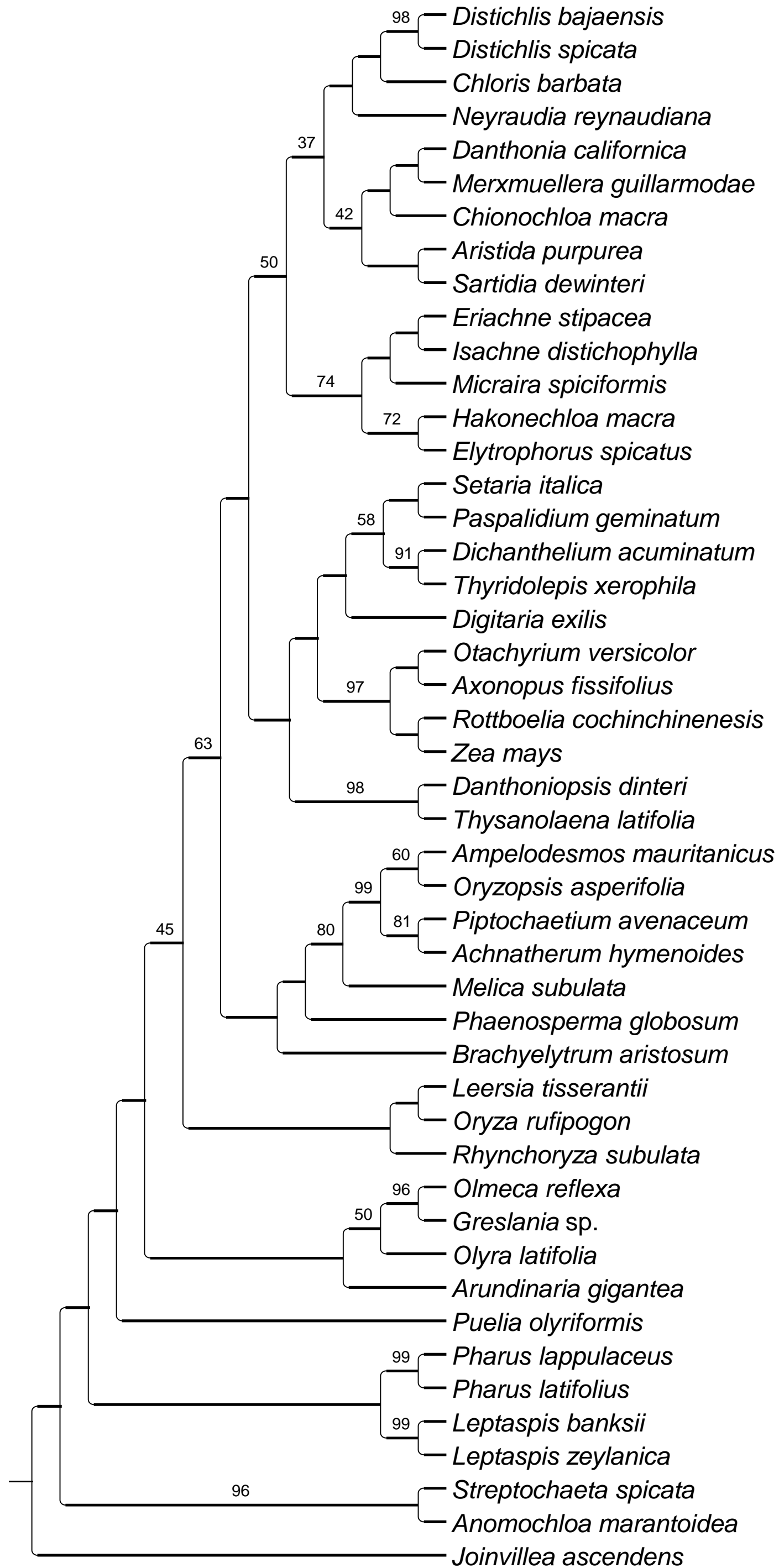

Supplemental 4: The ML tree from the two gene data set. All ML BV = 100% unless indicated.

Supplement: Supplementary file 4 [file Image_3.PDF]

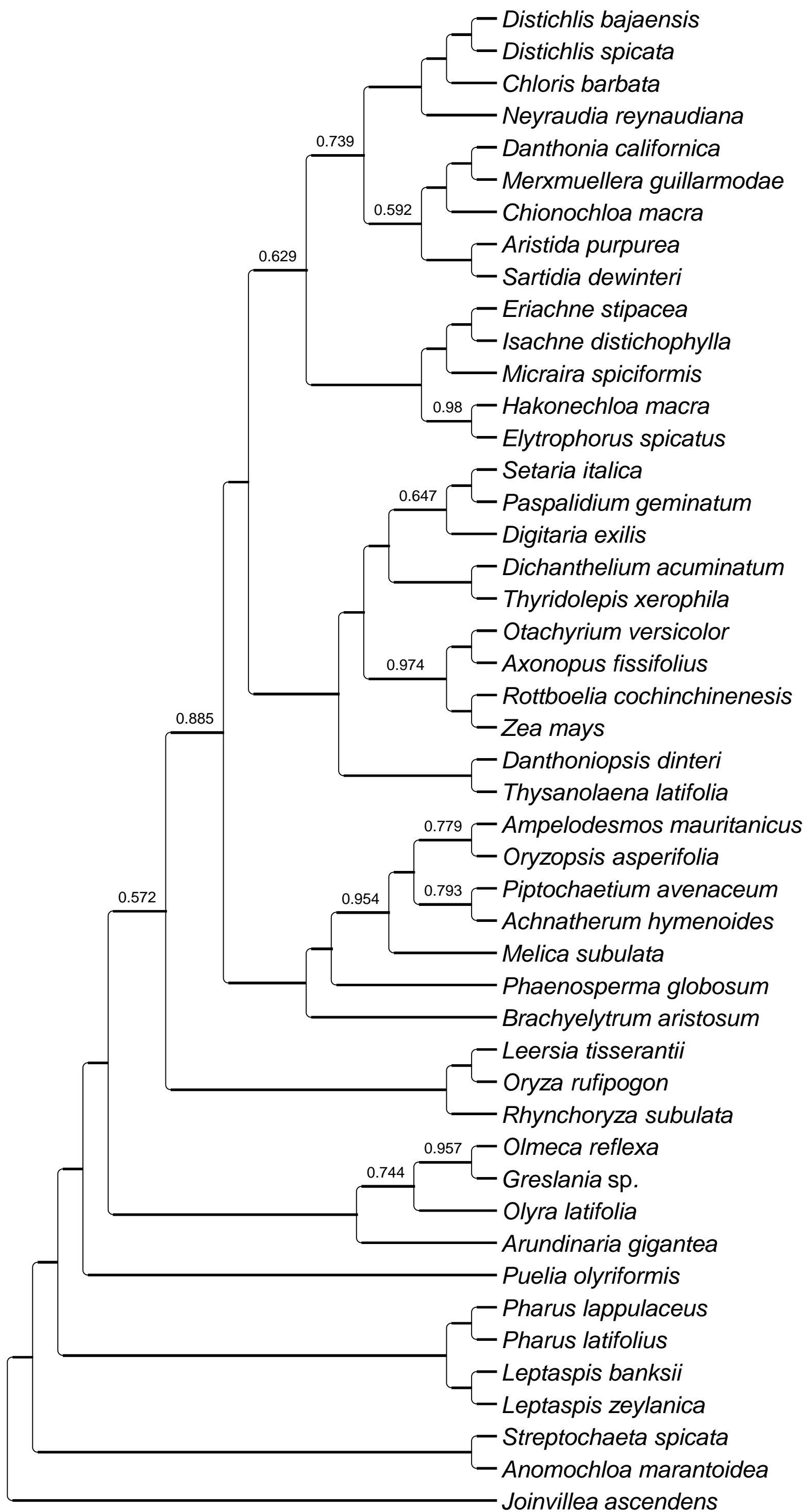

Supplemental 5: The BI tree from the two gene data set. All PP = 1.0 unless indicated.

Supplement: Supplementary file 5 [file Image_4.PDF]
